# Improved pain and joint function with absorbable pins and mini external fixator distraction in Freiberg disease: a 41-month follow-up study

**DOI:** 10.3389/fsurg.2025.1664036

**Published:** 2025-11-03

**Authors:** Zhibin Lai, Yuying Lin, Yongzhan Zhu, Kangyong Yang, Junhui Lai, Hongning Zhang, Baoli Zou, Chen Cheng, Guodong Shen

**Affiliations:** 1 The Eighth Clinical Medical College of Guangzhou University of Chinese Medicine, Foshan, Guangdong, China; 2 Department of Foot and Ankle Orthopedics, Foshan Hospital of Traditional Chinese Medicine, Foshan, Guangdong, China; 3 The Spinal Column Minimally Department, The Second Affiliated Hospital of Guangzhou University of Chinese Medicine, Guangzhou, China

**Keywords:** Freiberg disease, dorsal closing wedge osteotomy, absorbable pins, mini external fixator, joint distraction

## Abstract

**Purpose:**

Freiberg disease, a relatively uncommon condition affecting the metatarsal head, often requires surgical intervention when conservative treatments fail. Using absorbable pins combined with mini external fixator distraction, we aimed to assess the efficacy of dorsal closing wedge osteotomy (DCWO) in the treatment of Freiberg disease.

**Methods:**

This retrospective study analyzed data from 18 patients with Freiberg disease treated from June 2016 to June 2020. Patients were treated with DCWO using absorbable pins combined with mini external fixator distraction. Clinical and functional outcomes included Visual Analogue Scale (VAS) for pain, American Orthopaedic Foot & Ankle Society Lesser Metatarsophalangeal-Interphalangeal (AOFAS-LMI) score, and active sagittal-plane ROM of the second MTP measured with a specific goniometer. Radiologic assessment used weight-bearing AP foot radiographs to quantify second-metatarsal shortening (normalized to first-metatarsal length). The progression of MTP arthritis was a focus of assessment. Assessments were performed preoperatively, at 6 months, 1 year, and final follow-up. Complications were systematically documented.

**Results:**

Compared to preoperative values, significant improvements were observed in VAS scores, AOFAS-LMI scores, and metatarsophalangeal joint ROM over an average follow-up duration of 41.0 ± 17.0 (0.5–20) months. Specifically, VAS scores decreased from 5 (4, 5) to 0.5 (0, 1), while AOFAS-LMI scores increased from 68.3 ± 5.0 to 92.5 ± 2.9. Furthermore, dorsiflexion improved from (15.83 ± 6.4)° to (32.7 ± 4.8)°, plantarflexion increased from (13.9 ± 6.1)° to (29.3 ± 4.3)°, and the total ROM increased from (29.7 ± 9.0)° to (62.5 ± 6.9)°. All changes were statistically significant (*P* < 0.01). The average metatarsal shortening was 2.96 ± 0.70 (1.8–4.1) mm. The incidence of pin-related infections associated with external fixators was 5.6%. No radiographic or clinical evidence of osteoarthritis was observed in the metatarsophalangeal joint at the final follow-up.

**Conclusion:**

DCWO with absorbable pins and mini external fixator distraction is safe and effective for treating Freiberg disease. It enables early functional exercise, avoids the need for surgical removal of internal implants, and potentially delays the progression of arthritis with distraction techniques.

## 1 Introduction

Freiberg disease is the osteochondrosis of the metatarsal head, most commonly affecting the second metatarsal head. In the early stages, the disease presents with localized ischemia of the epiphysis, progressing to cancellous bone resorption, cartilage collapse, and finally arthritis (1). Localized pain at the affected metatarsal head, swelling of the surrounding tissues, and reduced range of motion are the primary symptoms (2, 3). Despite being first described over a century ago, the exact pathogenesis of Freiberg disease remains unclear. Mechanical stress, vascular damage, and genetic factors have been implicated in the pathogenesis of this disease. In particular, foot trauma, biomechanical abnormalities, excessive load on the metatarsal heads, and vascular damage leading to insufficient blood supply were shown to be involved in the development of Freiberg disease (4, 5). Furthermore, systemic diseases such as diabetes, systemic lupus erythematosus, and hypercoagulable states may predispose individuals to Freiberg disease by decreasing blood flow to the metatarsal head (3). One hypothesis (6, 7) is that repetitive microtrauma to the metatarsal head can lead to subchondral fractures, which subsequently disrupt blood supply, lead to avascular necrosis and subsequent collapse of the bone. Vascular compromise (8), whether due to systemic diseases or localized injury, may exacerbate these effects. Another theory suggests that anatomical variations, such as a longer second metatarsal bone (6, 9), may increase mechanical stress on the metatarsal head, making it more susceptible to injury.

The management of Freiberg disease typically begins with conservative treatments to alleviate the symptoms and prevent further articular damage. Conservative treatments include lifestyle modification, use of orthotics, physical therapy, and non-steroidal anti-inflammatory drugs (NSAIDs). However, surgical intervention becomes necessary when conservative measures fail to provide relief. The primary goals of surgical treatment are to alleviate pain, restore the function of metatarsophalangeal joint, and re-establish proper weight distribution across the foot. Currently, there are various surgical treatment options for Freiberg disease, including debridement, osteotomy, core decompression, microfracture, autologous osteochondral transplantation, joint replacement, etc. (10). Dorsal closing wedge osteotomy (DCWO) of the metatarsal head is widely used in clinical practice and is considered the gold standard treatment for Freiberg disease (11). Numerous studies (12–14) have reported favorable outcomes with this surgical technique.

However, certain issues in current therapeutic approaches remain inadequately addressed. First, Metallic screws or K-wires are commonly used to fix the osteotomy site in clinical practice; however, these methods have several disadvantages, such as metal irritation, implant breakage, stress shielding, implant migration, allergic reactions, tendinitis, and the need for a second surgery to remove the hardware (15, 16). Second, inadequate treatment may allow Freiberg disease to evolve into osteoarthritis (1, 17); moreover, arthritic deterioration has been observed even following seemingly appropriate surgical management. A prospective observational study (13) reported that 2.9% (1/34) of patients develop arthritis after dorsal metatarsal closing wedge osteotomy for treating Freiberg disease. Thus, it is critical to overcome the limitations of metal fixation and prevent postoperative progression to arthritis. Absorbable screws have been widely adopted as an alternative to metal fixation in orthopedic forefoot surgery. They may reduce metal-related complications, obviate the need for secondary implant removal (18, 19), and reduce imaging artifacts. Distraction arthroplasty is a therapeutic technique in which longitudinal traction is applied to separate the articular surfaces, thereby reducing intra-articular load, improving joint function, and facilitating tissue repair. This technique has been extensively applied in the treatment of ankle osteoarthritis and have demonstrated efficacy (20–22). Building on these findings, we investigated whether a combined approach—dorsal closing-wedge osteotomy (DCWO) of the metatarsal head with absorbable pin fixation plus joint distraction via a mini external fixator—could address the aforementioned challenges in Freiberg disease.

## 2 Methods

### 2.1 Patients

**Figure 1:**
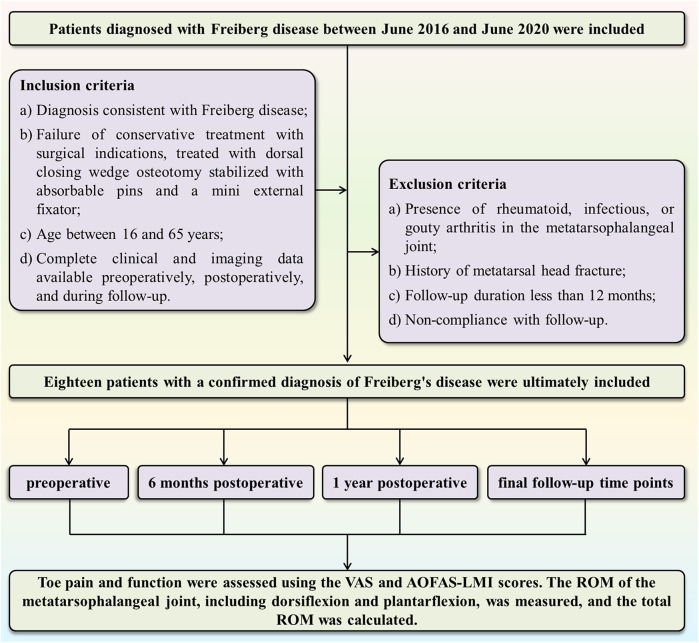
Study flowchart.

This study was a retrospective analysis that included 18 patients diagnosed with Freiberg disease at our department between June 2016 and June 2020. All patients had necrosis of the second metatarsal head and were treated with DCWO of the metatarsal head, using absorbable pins combined with a mini external fixator for intraoperative stabilization. Participants were screened based on the inclusion and exclusion criteria (Figure 1). Inclusion criteria were as follows: 1) Diagnosis consistent with Freiberg disease according to the combination of clinical symptoms and imaging examinations (10, 23): i. Patient presented with pain, swelling, tender to palpation and limited range of motion over the affected MTP joint; ii. Imaging findings, including flattening and sclerosis of the metatarsal head, joint space narrowing, and the presence of intra-articular loose bodies, were revealed on weight-bearing radiographs (AP, lateral, oblique) or CT scans. In selected atypical cases, MRI was obtained to identify occult lesions; 2) Patients who failed ≥6 months of conservative treatment (oral NSAIDs, activity modification, avoidance of high-impact exercises, joint immobilization, and custom orthotic devices) and met surgical indications underwent DCWO stabilized with bioabsorbable pins and a mini external fixator; 3) Age between 16 and 65 years; 4) For all patients, complete clinical records and imaging assessments—weight-bearing radiographs (anteroposterior, lateral, and oblique views) —were obtained preoperatively, postoperatively, and during follow-up evaluations. Additionally, computed tomography (CT) scans were obtained preoperatively to assess joint morphology. Exclusion criteria were as follows: 1) Presence of rheumatoid, infectious, or gouty arthritis in the metatarsophalangeal joint; 2) History of metatarsal head fracture; 3) Follow-up duration shorter than 12 months; and 4) Non-compliance with follow-up. This study was approved by our Hospital's Medical Ethics Committee (Ethics No: 2021082), it was conducted in accordance with the Declaration of Helsinki and informed consent was waived due to the retrospective nature of this study.

### 2.2 Surgical method

**Figure 2:**
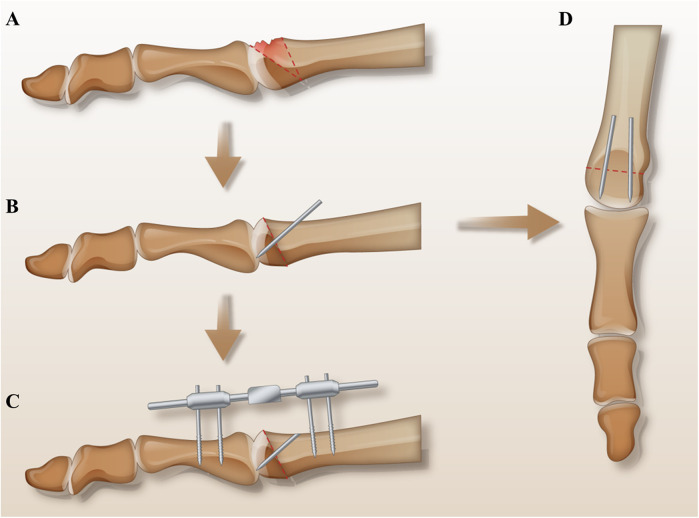
Surgical schematic diagram: **(A)** shows the necrotic area and the osteotomy line. **(B,D)** Demonstrate the dorsal closing wedge osteotomy, where the lesion was excised, the osteotomy site was closed, and the intact plantar joint surface was rotated dorsally. Two absorbable pins were inserted along the vertical osteotomy line. **(C)** Illustrates the installation of the mini external fixator.

**Figure 3:**
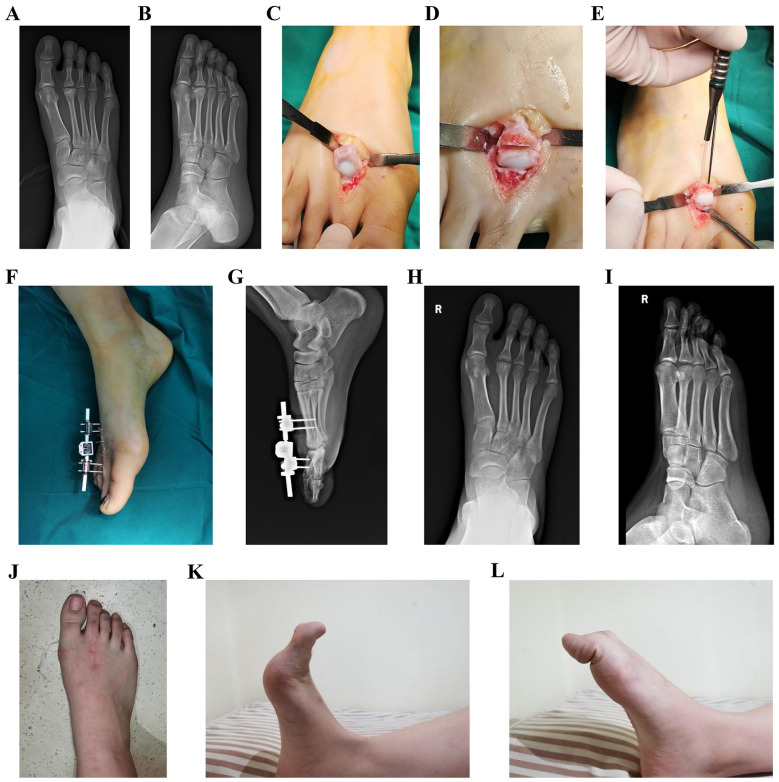
Typical cases. **(A,B)** Preoperative radiographs; **(C–E)** surgical procedure; **(F)** postoperative immobilization; **(G)** postoperative radiographs; **(H–L)** radiographs and activity at 28-month postoperative follow-up.

The patients were placed in a supine position under spinal anesthesia, with a tourniquet applied to the proximal thigh to control bleeding in the affected limb. A longitudinal incision approximately 4 cm in length was made over the dorsum of the second metatarsophalangeal joint, carefully protecting the nerves and tendons while retracting the tendons laterally. The joint capsule was incised to expose the metatarsophalangeal joint. Hypertrophic synovium was excised, and osteophytes and loose bone fragments in the joint cavity were removed from the second metatarsal head. Osteophyte hyperplasia at the base of the proximal phalanx of the second toe was also excised. The metatarsophalangeal joint was then flexed to expose the necrotic portion of the metatarsal head, and an intra-articular closing wedge osteotomy was conducted. The osteotomy was directed from the distal dorsal aspect to the proximal plantar aspect. The base of the wedge was determined based on the size of the necrotic area. The necrotic portion was removed, and the osteotomy was closed by rotating the intact plantar joint surface dorsally. Two 2 mm diameter K-wires were temporarily inserted from the proximal dorsal aspect to the distal plantar aspect to secure the osteotomy site and to guide the path for the absorbable pins. Thereafter, the K-wires were sequentially removed and replaced along the hole with two 2 mm × 30 mm absorbable pins [Biofix, the fourth generation of bioabsorbable memory screw, manufactured by Bioretec, Finland. ActivaPin™ Bioabsorbable Fixation Pin: Specifications: Diameter: 2 mm; Length: 20–70 mm; Material: Poly(lactic-co-glycolic acid) (PLGA); Mechanical Properties: provides stable fixation strength for more than 16 weeks, and fully resorbed approximately 2 years after implantation. Design Features: the pin surface features a grooved structure, enabling a self-locking mechanism.), which were cut flush with the bone surface. Subsequently, to reduce mechanical stress on the articular cartilage and create a favorable environment for cartilage repair—thereby minimizing the occurrence and progression of postoperative arthritis—a mini external fixator was applied to achieve joint distraction. A mini external fixator (Tianjin Xinzhong Medical, China, L = 80 mm, external fixation pins Φ = 2.0 mm) was then assembled. Two partially threaded pins were percutaneously inserted into the metatarsal shaft and the proximal phalanx shaft, and the pin clamps were adjusted to maintain a distance of 5–10 mm from the skin. Subsequently, the clamps were tightened. The main body of the fixator with dual ball joints was attached, and the metatarsophalangeal joint was distracted to a gap of 5 mm, ensuring proper joint alignment and mechanical axis before tightening the lateral knobs and ball joint knobs. The external portions of the pins were trimmed, leaving 2 mm outside the clamps. Partially threaded pins were inserted perpendicular to the bone, avoiding damage to the extensor tendons. The surgical field was irrigated, and the incision was closed (Figures 2, 3).

### 2.3 Postoperative management and follow-up

Prophylactic antibiotics were administered for 24 h after the surgery. Special attention was paid to postoperative toe sensory function as part of our routine safety protocol. Neurovascular checks (light-touch/pin-prick sensation, capillary refill, skin temperature) were performed at regular intervals. If any new numbness or paresthesia suggestive of nerve tension had been detected, the distraction would have been immediately reduced by adjusting the external fixator (rather than removing it), and the remaining distraction would have been resumed gradually under inpatient observation.. On the third postoperative day, the central dual ball joint knob of the external fixator was loosened to allow for flexion-extension exercises of the metatarsophalangeal joint without altering the distraction length. Two weeks after the surgery, patients were allowed to bear weight while walking with a forefoot offloading shoe. The pin sites were kept clean and dry, with regular antiseptic dressing changes. Follow-up visits were conducted in the outpatient clinic at 4, 8, and 12 weeks, 6 months, and 1 year postoperatively, and at the final follow-up, during which foot x-rays were taken. The external fixator was removed in the outpatient clinic 6 weeks after the surgery. Based on bone healing, a gradual transition to full weight-bearing with normal shoes was initiated between 8 and 12 weeks after the surgery.

### 2.4 Efficacy evaluation

**Figure 4:**
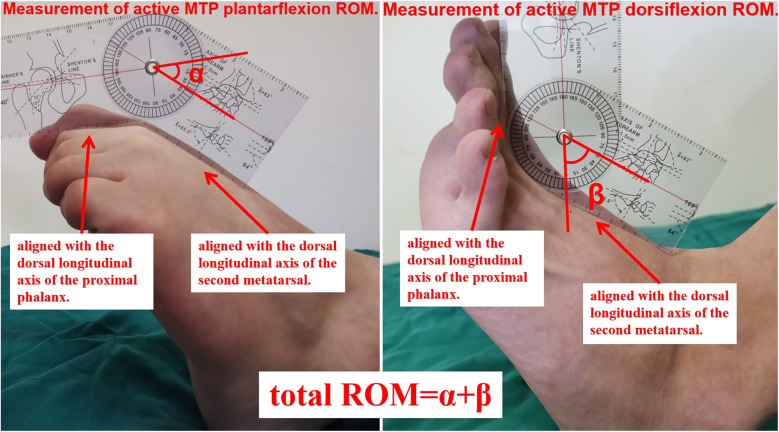
Measurement of metatarsophalangeal joint range of motion (ROM). With the patient supine or seated and the foot and ankle in neutral alignment, the goniometer fulcrum was positioned over the dorsal center of the second MTP joint. The proximal arm of the goniometer was aligned with the dorsal longitudinal axis of the second metatarsal, and the distal arm with that of the proximal phalanx. Patients were then instructed to actively move to end-range plantarflexion (α) and dorsiflexion (β), at which points the angles were recorded. Total ROM = *α* + *β*.

**Figure 5:**
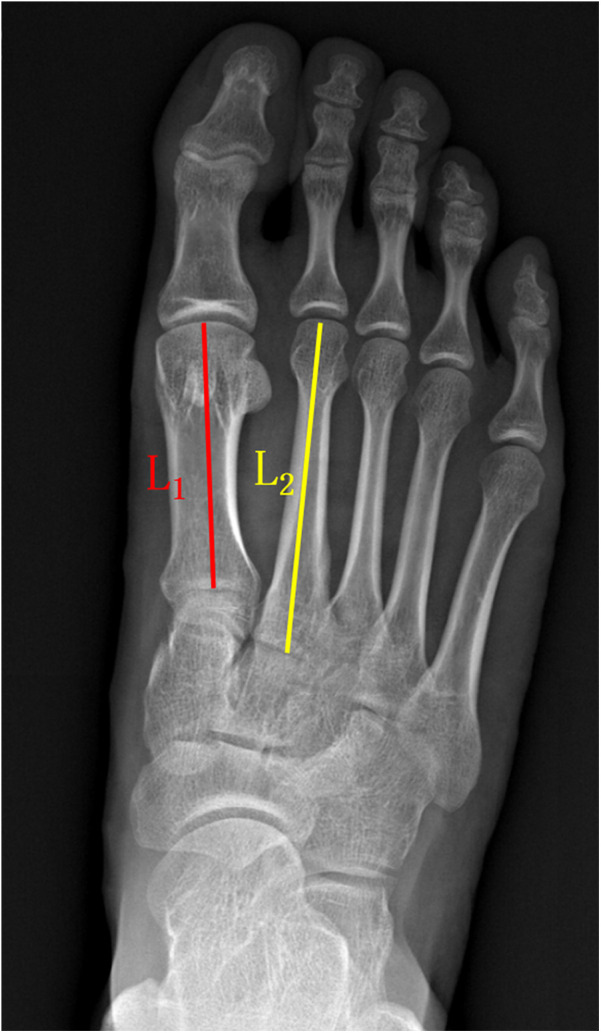
The method used for measuring metatarsal bone length. The preoperative length was used as the reference standard. The length of the first metatarsal bone (L1 pre) and the second metatarsal bone (L2 pre) was measured before the surgery. At the final follow-up, the length of the first metatarsal bone (L1 post) and the second metatarsal bone (L2 post) was measured. The magnification factor for L2 post was calculated as L1 post/L1 pre, and the change in length for L2 (L0) was calculated as L0 = L2 post/(L1 post/L1 pre) - L2 pre.

VAS (a numerical scale with a range from 0 to 10, where 0 represented “no pain” and 10 indicated “worst possible pain.”) and AOFAS-LMI scores were used to assess toe pain and function preoperatively and at 6 months postoperative, 1 year postoperative, and the final follow-up. The ROM of the metatarsophalangeal joint, including dorsiflexion and plantarflexion, was measured, and the total ROM was calculated. We measured active sagittal-plane range of motion (dorsiflexion and plantarflexion) of the second metatarsophalangeal (MTP) joint using a flexible, MTP-specific goniometer. With the patient supine or seated and the foot and ankle in neutral, the goniometer fulcrum was placed over the dorsal center of the second MTP joint. The proximal arm was aligned with the longitudinal axis of the second metatarsal, and the distal arm with the proximal phalanx. Patients were then instructed to actively move to end-range dorsiflexion and plantarflexion, at which point the angle was recorded (Figure 4). Metatarsal shortening was measured on weight-bearing anteroposterior x-rays of the foot using the following method. The preoperative length of the first metatarsal (L1 pre) and the second metatarsal (L2 pre) were measured using the first metatarsal length as a reference. At the final follow-up, the lengths of the first metatarsal (L1 post) and second metatarsal (L2 post) were measured, and the change in length (L0) was calculated as follows: L0 = L2 post/(L1 post/L1 pre) - L2 pre (Figure 5). Related complications (non-union, osteomyelitis, necrosis of the metatarsal head, or progression of metatarsophalangeal joint arthritis) were recorded.

### 2.5 Statistical methods

Statistical analysis was conducted using SPSS 25.0 software. Continuous variables are presented as mean ± standard deviation (X ± s). Regarding normally distributed data, comparisons between different time points within the same group were conducted using paired *t*-tests or one-way analysis of variance (ANOVA). Concerning non-normally distributed data, paired comparisons were made using the Wilcoxon signed-rank test. Categorical data were analyzed descriptively or presented as percentages (%). *P*-values less than 0.05 were considered statistically significant.

## 3 Results

### 3.1 Clinical outcomes

In total, there were 9 patients with left side disease and 9 patients with right side disease. Of them, 16 (88.9%) were females and 2 (11.1%) were males. Based on Smillie's classification (10), 8 patients were classified as stage III, 9 patients as stage IV, and 1 patient as stage V. Patients' ages ranged from 17 to 57 years (33.0 ± 11.7), and their disease duration ranged from 0.5 to 20 years (4.4 ± 4.4 years). Five patients (27.8%) had a history of trauma (all mild trauma, including 2 sprains or strains of the foot, 1 contusion, 2 minor falls or twists), while the remaining patients had no clear history of trauma or were unable to recall any prior traumatic events. No patient experienced postoperative toe numbness, dysesthesia, or ischemic changes, and no early reduction of distraction or unplanned adjustment was required. The follow-up period ranged from 18 to 72 months, with an average of 41.0 ± 17.0 months (Table 1). Compared to preoperative values, there were significant improvements in VAS and AOFAS-LMI scores at the final follow-up (*P* < 0.05). Additionally, dorsiflexion, plantarflexion, and total ROM of the metatarsophalangeal joint significantly improved compared to preoperative measurements (*P* < 0.05) (Table 2). There were no cases of neurovascular injury, transfer metatarsalgia, implant rejection, or irritation. One patient (5.6%) underwent wound drainage and peripheral redness at the pin site three weeks after the surgery, which resolved after removing the external fixator and wound care. No other pin-site infections, deep infection or osteomyelitis were noted.

**Table 1:** Demographic data of 18 patients treated for Freiberg disease.

Variable	Numerical value
Age [mean ± SD (years)]	33.0 ± 11.7 (17–57)
Disease duration [mean ± SD (years)]	4.4 ± 4.4 (0.5–20)
Gender	
Male	2 (11.1%)
Female	16 (88.9%)
Affected side	
Right	9 (50.0%)
Left	9 (50.0%)
Smillie's classification	
Stage III	8 (44.4%)
Stage IV	9 (50.0%)
Stage V	1 (5.6%)
History of trauma	
Yes	5 (27.8%)
No	13 (72.2%)
Follow-up period [mean ± SD (months)]	41.0 ± 17.0 (0.5–20)

L, left; R, right; SD, standard deviation; Y, years; M, months.

**Table 2:** Comparison of preoperative and postoperative clinical follow-up results in 18 patients.

Evaluation indicator	Before the surgery	6 months postoperative	1 year postoperative	Final follow-up	Statistical value	*P*
VAS [Scores, median (quartile)]	5 (4, 5)	2 (1, 2)*	1 (0, 1)*	0.5 (0, 1)*	45.4	<0.01
AOFAS (Scores, X ± s)	68.3 ± 5.0	86.4 ± 4.6*	90.5 ± 2.4*	92.5 ± 2.9*	145.3	<0.01
Dorsiflexion ROM (°, X ± s)	15.83 ± 6.4	26.8 ± 4.5*	28.1 ± 3.7*	32.7 ± 4.8*	29.3	<0.01
Plantarflexion ROM (°, X ± s)	13.9 ± 6.1	21.7 ± 6.3*	25.4 ± 4.8*	29.3 ± 4.3*	19.6	<0.01
Total ROM (°, X ± s)	29.7 ± 9.0	48.5 ± 7.2*	53.5 ± 5.4*	62.5 ± 6.9*	49.4	<0.01

* Compared to before the surgery, the difference was significant (*P* < 0.05). X ± s, mean ± standard deviation.

### 3.2 Radiographic evaluation

Weight-bearing anteroposterior x-rays of the foot showed a normal joint space in the second metatarsophalangeal joint at the final follow-up, with good alignment and no signs of dislocation or subluxation. All patients exhibited bony union at the osteotomy site, with no complications, such as non-union, osteomyelitis, necrosis of the metatarsal head, or progression of metatarsophalangeal joint arthritis. The shortening of the second metatarsal ranged from 1.8 mm to 4.1 mm, with an average of 2.96 ± 0.70 mm.

## 4 Discussion

In this study, DCWO combined with absorbable pins and mini external fixator distraction was applied to treat 18 patients with Freiberg disease, and favorable outcomes were obtained. At the final follow-up, the VAS and AOFAS-LMI scores significantly improved, with marked enhancements in dorsiflexion, plantarflexion, and total ROM of the metatarsophalangeal joint. There were not any cases of neurovascular injury, implant rejection, or irritation, except for one case of pin-site infection, which resolved with treatment. Weight-bearing x-rays showed normal joint space and alignment in the affected metatarsophalangeal joint, with no dislocation or subluxation. All patients achieved bony union. The minimal shortening of the second metatarsal did not bring transfer metatarsalgia as well. Most importantly, no evidence of metatarsophalangeal (MTP) joint arthropathy was observed at the final follow-up.

DCWO is highly effective in managing Freiberg disease, which can significantly improve patients' outcomes. This improvement can be attributed to the ability of osteotomy to shorten the metatarsal bone, elevate the metatarsal head, and rotate the intact plantar joint surface, all of which reduce joint overload and reconstruct the joint. The procedure not only relieves pain but also rebalances the forces across the metatarsophalangeal joint, thereby restoring its function and ensuring long-term joint stability. Georgiannos et al. *(*11) reported that 14 patients with Freiberg disease were treated with DCWO surgery. The osteotomy site was fixed with 2 - 0 nonabsorbable sutures. After 3 years of follow-up, the AOFAS-LMI scores increased from 63.0 before surgery to 84.4 (*P* < 0.05), and the visual analog scale-foot and ankle score improved significantly from 48.1to 91.8. Kim et al. (24) treated 15 late-stage Freiberg disease cases with DCWO fixed by one mini-screw. Over an average 38.7-month follow-up, AOFAS-LMI rose from 70.5 to 87.9 and VAS declined from 6.9 to 1.9 (*P* < 0.05). In a cohort of 34 patients with Freiberg disease, Öztürk et al. (13) performed dorsal closing-wedge osteotomy (DCWO) stabilized with a single 2.5-mm cannulated headless compression screw. At a mean follow-up of 33.7 months, the mean AOFAS score improved from 53.24 to 86.26 (*P* < 0.05), while the mean VAS score declined from 8.59 to 1.79 (*P* < 0.05). In a multicenter series (14), eight patients with Freiberg disease underwent dorsal closing-wedge osteotomy (DCWO). Fixation was achieved with two crossing Kirschner wires, which were removed at six weeks. Over a mean follow-up of 37 months, AOFAS-LMI scores increased from 58.88 to 86.75 (*P* < 0.05), while VAS scores declined from 6.75 to 2.0 (*P* < 0.05). The studies utilized heterogeneous internal fixation methods, resulting in correspondingly variable outcomes. In our cohort (*n* = 18), AOFAS-LMI improved from 68.3 preoperatively to 92.5 at final follow-up (mean change 24.2 points; within-group *P* < 0.001). This magnitude of improvement is consistent with those reported above, suggesting that this technique may be comparably effective.

There are several advantages using absorbable pins combined with mini external fixator distraction. First, absorbable pins prevent complications associated with metallic implants, such as irritation, breakage, infection, and the need for removal surgery (7). Helix-Giordanino M et al. (25) treated 30 patients with Freiberg disease using dorsal closing-wedge osteotomy fixed by one or two thin metal staples, and 5 patients (16.7%) experienced foot discomfort due to metallic implants, with 3 cases (10.0%) requiring removal of the hardware. These absorbable pins provide strong fixation support in the early postoperative period, creating a stable mechanical environment for healing (19). With their degradation, stress gradually transfers to the healing bone, promoting bone growth and preventing stress shielding. Eventually, the pins are fully absorbed and replaced with new bone, avoiding the need for surgical removal of internal implants such as screws. This approach decreases the risks associated with metallic implants and facilitates a natural healing process. Second, distraction arthroplasty has been widely employed in the management of ankle osteoarthritis and has demonstrated clinical efficacy (20–22). In our study, the mini external fixator offers joint distraction, which reduces mechanical stress on the joint cartilage, improves cartilage nutrition through intermittent fluid pressure, and reduces subchondral sclerosis, thereby forming a favorable environment for cartilage healing (22, 26, 27). A prospective study (13) reported a 2.9% (1/34) incidence of postoperative arthritis following DCWO, indicating that arthritic progression remains possible after joint-sparing procedures. In our study, no evidence of arthritis in the metatarsophalangeal joint was observed at the final follow-up. This effect may result from the joint distraction achieved by the use of a mini external fixator. These cross-study observations are contextual and do not allow comparative inference; our findings should be considered hypothesis-generating. Third, the dual ball joint structure of the fixator allows early flexion-extension exercises, contributing to early rehabilitation while maintaining joint distraction, preserving joint mobility, and promoting rapid recovery. Early mobilization promotes circulation, reduces stiffness, and expedites recovery, making it a critical component of postoperative care. Incesoy MA et al. (28) performed DCWO for eight Freiberg disease cases, with the osteotomy stabilized by two crossed K-wires. At a mean follow-up of 40 months, dorsiflexion improved from 10° to 29.38°; however, plantarflexion declined from 19.38° to 6.88°. In an additional cohort (29) treated with K-wire osteotomy fixation, the average restrictions in passive flexion and extension measured 18° and 12°, respectively. According to Helix-Giordanino M et al. (25), DCWO stabilized with a staple was associated with average decreases of 15° in plantarflexion and 10° in dorsiflexion at 6.5 years of follow-up. In the aforementioned series where postoperative ROM decreased, fixation was generally achieved with K-wires or staples; these devices do not allow early mobilization, which largely explains the observed loss of motion, with soft-tissue irritation as a possible adjunct factor. In our cohort, the range of motion increased from 29.7° preoperatively to 62.5° at the final evaluation. Improvement in mobility is plausibly attributable to the mini external fixator, which preserves osteotomy stability while permitting early, controlled flexion–extension rehabilitation. Furthermore, temporary joint distraction increases intra-articular space, a mechanism that may mitigate capsular contracture and consequently diminish postoperative stiffness.

A potential complication of DCWO is excessive metatarsal shortening, which can result in floating toe and transfer metatarsalgia in severe cases. However, moderate shortening is generally safe. Lee et al. (30) reported an average shortening of 3.2 mm with no cases of transfer metatarsalgia (*n* = 15). In this study, the average shortening was 2.96 ± 0.70 mm without transfer metatarsalgia (*n* = 18), with no other complications, highlighting the importance of careful intraoperative management. The precise control of the osteotomy angle and bone resection is pivotal to prevent excessive shortening. Excessive shortening can alter foot biomechanics and lead to pain in adjacent metatarsals. Intraoperative fluoroscopy provides a real-time guide to optimize the procedure.

This technology also has some drawbacks. First, superficial pin-site infection is a recognized issue associated with external fixation. In our series, the incidence was 5.6% (1/18), and the case was low-grade and resolved with removing the external fixator and wound care, without deep infection or osteomyelitis. These findings reflect the importance of standardized pin care and early surveillance, and they should be considered when weighing the trade-offs of incorporating a mini external fixator into a protocol. Second, it is important to clarify that while the use of absorbable pins eliminates the need for implant removal, the mini external fixator employed in this technique still requires a scheduled removal procedure, typically performed in the outpatient clinic without anesthesia. Although minimally invasive, this step represents an additional intervention compared to internal fixation methods using only screws. Therefore, while the technique reduces the burden of hardware-related complications, it does not fully eliminate the need for postoperative device management. This distinction should be acknowledged when evaluating its overall advantages. Third, Compared to metallic screws, absorbable screws are relatively more expensive and may carry device-specific risks, including rare allergic or foreign-body reactions; such events have been reported in the orthopedic literature (31). No allergic reactions were observed in our case series.

This study has several limitations, including a relatively small sample size, the absence of a control group, and the fact that it was a retrospective cohort study. These limitations are largely due to the low incidence of Freiberg disease. The small sample size, while reflecting the rarity of the condition, limits the generalizability of our findings. Without a control group, it is challenging to attribute improvements solely to the intervention, as other factors such as the natural progression of the disease or placebo effects could affect the results. Future studies should address these shortcomings by conducting multi-center, large-sample, randomized controlled trials. These studies can provide higher-level evidence and help confirm our findings. Additionally, future studies should explore the long-term outcomes of different surgical techniques and investigate new treatment modalities, including biologics and regenerative therapies, to optimize the management of Freiberg disease. There is also a need for studies that compare the outcomes of different surgical techniques in treating Freiberg disease. Such comparative studies can help identify the most effective approaches and guide clinical decision-making. Moreover, future studies should explored the role of patient-specific factors, such as age, activity level, and comorbid conditions in determining the best surgical approach. Personalized treatment plans that take these factors into account can lead to better outcomes and patient satisfaction.

## 5 Conclusion

In conclusion, the use of DCWO combined with absorbable pins and mini external fixator distraction may provide satisfactory joint motion and functional outcomes with low complication rates in certain stages of Freiberg disease. This approach allows for early functional exercise, potentially slows the progression of metatarsophalangeal joint arthritis with distraction techniques, and avoids the need for a secondary surgical procedure to remove internal fixation devices. Postoperative outcomes are highly satisfactory, with minimal complications and good recovery of foot function. However, it should also be noted that, as with all external fixation devices, pin tract infections are a common and often unavoidable complication. In addition, the mini external fixator used in this technique requires scheduled removal, which constitutes an additional procedural intervention. This distinction should be taken into account when evaluating the overall benefits of the technique. Therefore, more and further studies with long-term follow-up are needed to validate these findings, fully assess its effectiveness and explore additional treatment options for this challenging condition.

## Data Availability

The original contributions presented in the study are included in the article/Supplementary Material, further inquiries can be directed to the corresponding author.
